# Data on network of live cattle exports from Brazil

**DOI:** 10.1016/j.dib.2018.06.059

**Published:** 2018-06-22

**Authors:** Marcos Eielson Pinheiro de Sá, Cláudia Valéria G.C. de Sá, Rafael R. Nicolino, João Paulo A. Haddad, Concepta McManus, Luiza Seixas, Cristiano Barros de Melo

**Affiliations:** a University of Brasília (UnB/PPGCA), Campus Darcy Ribeiro, ICC Sul, Asa Norte, Brasília, DF 70910-900, Brazil; b Ministry of Agriculture, Livestock and Food Supply (MAPA), Esplanada dos Ministérios, Brasília, DF, Brazil; c Federal University of Minas Gerais (UFMG/EV), Pampulha, Belo Horizonte, MG, Brazil

## Abstract

This report describes the network of live cattle exports from Brazil using Microsoft Office Excel® files, Terraview®, Maporama®, Pajek® and Google Maps® softwares. The database contains estimates obtained from the Ministry of Agriculture, Livestock and Food Supply (MAPA) and underwent descriptive, spatial and flow network. The network of live cattle exports from Brazil was determined using data from 27,517 Animal Transit Certificates (ATC) and 579 Veterinary Certificate for International Trade. International departure points, municipalities and states of origin, destination countries, purpose of export and compliance with sanitary requirements for exports, cattle movement and the main transportation corridors were showed through flow network. The states that exported live cattle were Pará, Rio Grande do Sul, Tocantins, São Paulo, Minas Gerais and Maranhão. Vila do Conde Port, located in Barcarena municipality in the state of Pará, was the main international departure point of animals, which were intended mostly for immediate slaughter in the importing country. The internal cattle transportation corridors of the main counties and farms that provide animals for exports in 2009 were mapped.

**Specifications Table**

**Table:** 

Subject area	Veterinary
More specific subject area	Cattle transit data
Type of data	Table, graph, figure
How data was acquired	Data bank - The network of live cattle exports from Brazil was determined using data from Animal Transit Certificates and Veterinary Certificates for International Trade
Data format	Analyzed
Experimental factors	Data was collected and compiled to produce maps of network of live cattle exports from Brazil in 2009.
Experimental features	Network maps were built using Microsoft Office Excel® files, Terraview®, Maporama®, Pajek® and Google Maps® softwares.
Data source location	Ministry of Agriculture, Livestock and Food Supply (MAPA) - Brazil
Data accessibility	The data is with the article

**Value of the data**

• Information on animal movement aids in understanding the paths that can be traversed by pathogens through direct or indirect contact with animals.
• Network analysis of animal movement is an useful tool for identifying important links in the epidemiological chain and assisting in the establishment of surveillance measures for prevention and control of diseases.
• They can be used to estimate the risk for animal diseases dissemination, the economic corridors for international cattle transportation and market analysis for cattle business.

## 1 Data

The data showed here was collected individually in the main international departure points of live cattle in Brazil in the year of 2009. Vila do Conde Port (Barcarena city, Pará state - PA), Rio Grande Port in Rio Grande do Sul state (RS) and São Sebastião Port in São Paulo state (SP), as well as through Viracopos International Airport (VCP) in Campinas (SP) were the international departure points studied in the present research. A total of 27,517 Animal Transit Certificates (ATC) and 579 Veterinary Certificates for International Trade were collected. Raw data regarding live cattle export can be found in Excel files (1 File Export and 2 File Export). Data of the network of live cattle exports from Brazil is also presented in this work using Terraview®, Maporama®, Pajek® and Google Maps® softwares in order to identify the countries that import animal commodities from Brazil, the main international departure points, municipalities and states of origin of the animals. Moreover, the logistics of trade corridors, social flow networks, distances and main corridors are presented.

## 2 Experimental design, materials and methods

### 2.1 Processing

The year of 2009 was randomly chosen as a model and represented a scenario after the global economic crisis that began in 2008 in the United States of America (USA) [1]. Network of live cattle exports from Brazil was determined using data from Animal Transit Certificates (ATC) and Veterinary Certificates for International Trade. Descriptive data of international departure points, destination countries, origin farms, states and municipalities of origin of the animals, as well as number, sex, purpose for exportation and compliance with sanitary requirements for exports was carried out. Raw data of Animal Transit Certificates and Veterinary Certificates for International Trade can be found in Microsoft Office Excel® (1 File Export and 2 File Export).

Thematic maps created using Terraview® 4.2.0 software [2] from the Brazilian Institute of Geography and Statistics (IBGE) aided in the spatial analysis of the live cattle transportation within the country, including information about the location of counties and farms, the MAPA System units for export, as well as the number of animals from each farm. The geographic coordinates of the centroid of municipalities, farms of origin and international departure points were determined using Maporama® Solutions (TIBCO Software Inc., Palo Alto - CA). The movement and number of cattle exported through ports and airports were established using Pajek® 6.4 Software [3].

Vila do Conde Port (Barcarena city, Pará state) is the main departure point from Brazil. Therefore, export data of this port was used to determine the major national transportation corridors for live cattle exports from Brazil. The flow corresponding to each corridor was estimated using Google Maps® (Google Inc, Mountain View, CA) and the municipality declared on the ATC data was considered as the origin and Barcarena the destination. The estimated distances, modes of transportation available and shortest corridors recommended by the major state and federal highways were recorded in Microsoft Office Excel® (Microsoft Corporation Office Excel, Portland) spreadsheets for further studies. Calculations of average, minimum and maximum distances and analysis of variance to compare average distances travelled by animals from each state were performed using the Statistical Analysis System 9.2 (SAS Institute, Cary, North Carolina) program.

Live cattle exports from Brazil occurred through the ports of Vila do Conde, in Barcarena – Pará state (PA), Rio Grande in Rio Grande do Sul state (RS) and São Sebastião in São Paulo state (SP), as well as through Viracopos International Airport (VCP) in Campinas, SP. Destination countries included Angola, Venezuela, Lebanon and Egypt, using mainly naval transport, with the exception of a few transactions through VCP to Venezuela.

A total of 513,319 live cattle was exported by Brazil in 2009. The Vila do Conde Port was the main international departure point with 493,984 (96.2%) animals from the state of Pará (PA), 3778 (0.7%) from Tocantins state and 95 (0.02%) from Maranhão state. Rio Grande Port exported 13,638 (2.7%) animals from Rio Grande do Sul, while the São Sebastião Port exported 1069 cattle (0.2%) from the state of São Paulo. A total of 755 (0.1%) animals from Minas Gerais (MG) went through VCP. Cattle were exported from 1697 farms located in 133 different municipalities. Of these, 1441 (84.9%) were located in Pará, 236 (13.9%) in Rio Grande do Sul, 12 (0.7%) in Tocantins, six (0.4%) in Maranhão, one (0.05%) in São Paulo and one (0.05%) in Minas Gerais (Table 1).

**Table 1:** Brazilian states, number of farms, municipalities, quantity and purpose of live cattle export in 2009.

**State**	**Farms**	**Municipalities**	**Number of cattle**		**Purpose**	
			**Males**	**Females**	**Slaughter**	**Reproduction**
São Paulo	1	1	99	970		1,069
Minas Gerais	1	1	31	724		755
Rio Grande do Sul	236	42	13,590	48	13,638	
Pará	1,441	80	493,984		496,990	
Tocantins	12	9	3,778			867
Maranhão	6	1	95			
**Total**	**1,697**	**133**	**511,577**	**1,742**	**510,628**	**2,691**

Of the cattle exports, 511,577 (99.7%) were males and 1742 (0.3%) females. Angola and Venezuela were the main destinations of exported females with 970 (55.7%) and 724 (41.6%) animals, respectively, they were loaded on São Sebastião Port and VCP Airport respectively. In addition, 48 (2.7%) females were imported by Lebanon from the Rio Grande Port, but no females were exported through the Vila do Conde Port during the studied period (Table 1).

As to the purpose of export, 510,628 (99.5%) animals were destined for immediate slaughter, of which 13,638 (2.7%) were loaded on Rio Grande Port for Lebanon and 496,990 (97.3%) on Vila do Conde Port for Lebanon, Egypt and Venezuela. In addition, 2691 (0.5%) were for reproduction, of which 1069 (39.7%) were loaded on São Sebastião Port and 755 (28.1%) on VCP, both had Angola as destination, while 867 (32.2%) cattle were loaded on Vila do Conde Port for Venezuela.

**Fig. 1:**
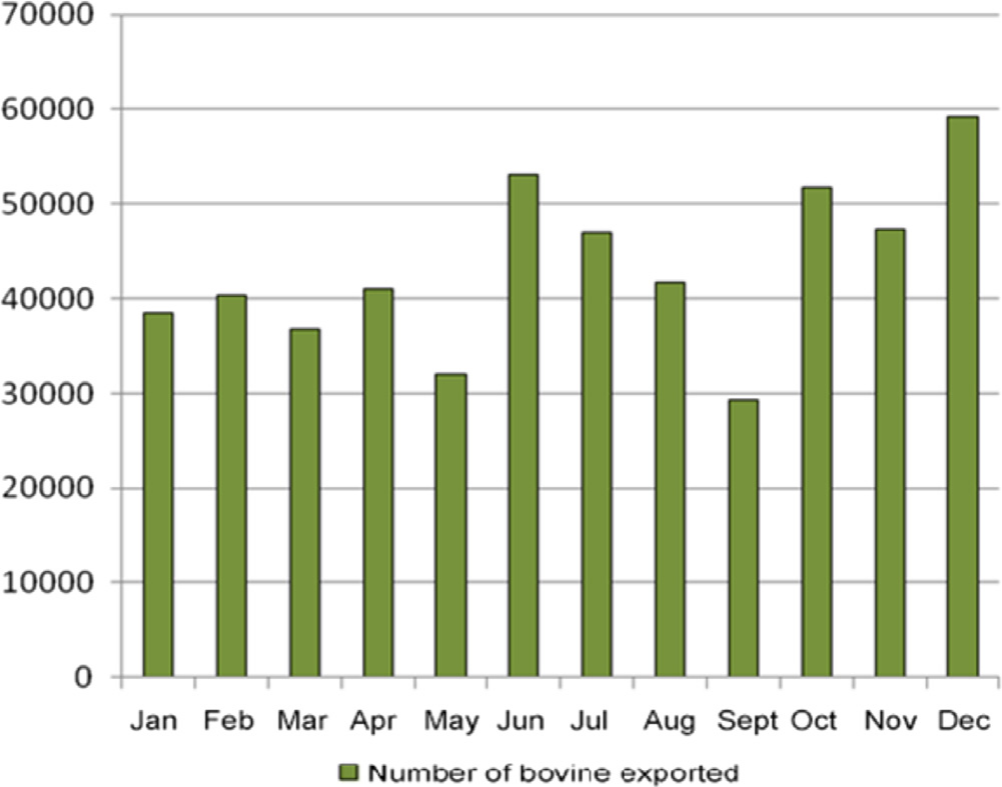
Number of live cattle export from Brazil in 2009 per month.

The monthly distribution of live cattle export from Brazil is shown (Fig. 1). Higher exports were seen between the months of June and July and from October to December 2009. Cattle exported through the Vila do Conde Port were from 1459 farms located in 90 municipalities, and 88.9% (80) were located in Pará state, nine (10%) in Tocantins state (TO) and one (1.1%) in Maranhão State (MA) (Table 1). In Pará state (PA), the counties with highest numbers of farms with cattle for export were Novo Repartimento and Paragominas (117 farms each) as well as Rondon do Pará (89 farms). In Tocantins and Maranhão states, the municipalities of Araguaína (two farms) and Açailândia (four farms) were the main suppliers of animals.

**Fig. 2:**
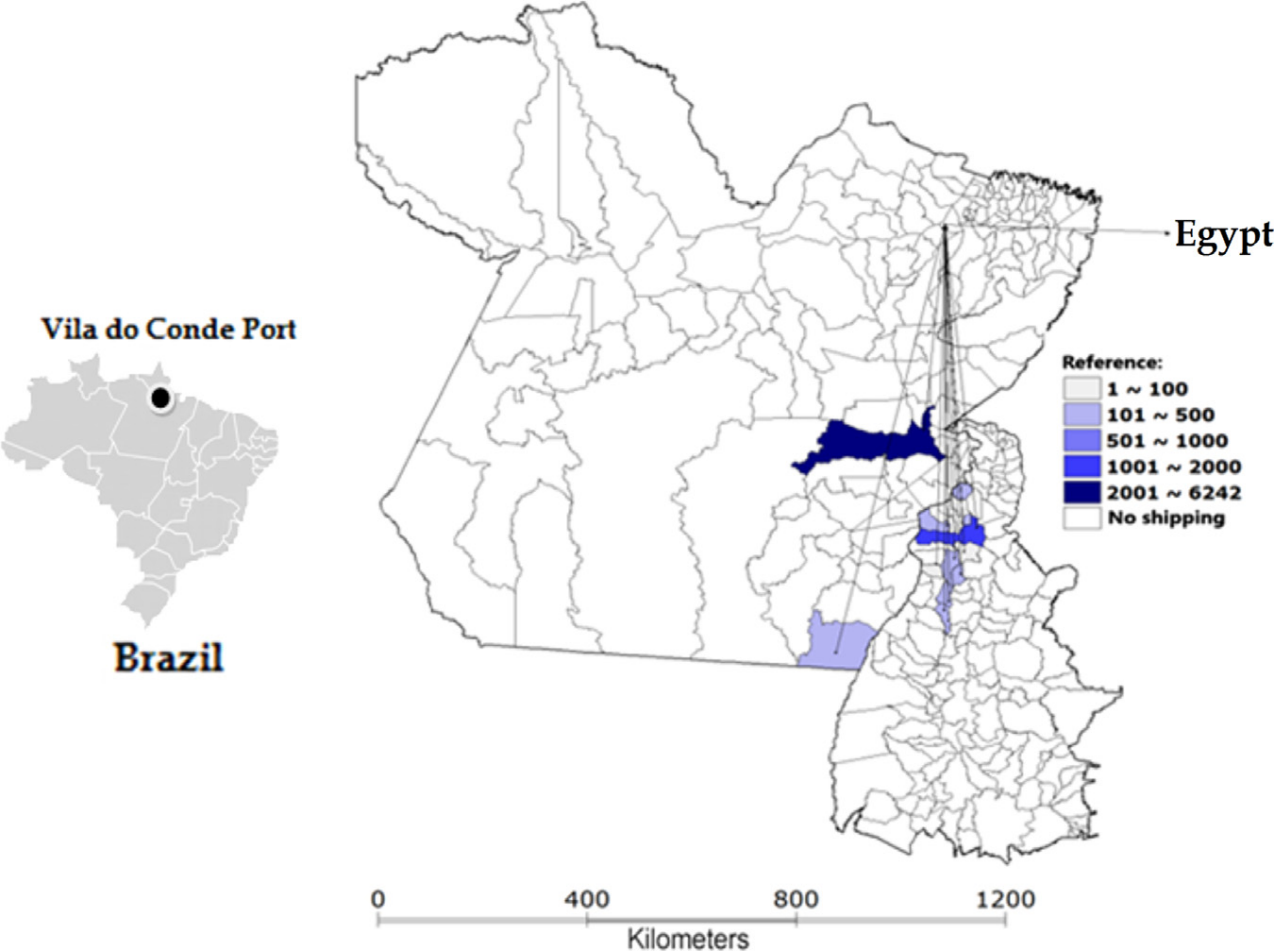
Flow of live cattle exports from Vila do Conde Port to Egypt, by municipality and number of animals, from Pará and Tocantins states in 2009 (with adaptations).

**Fig. 3:**
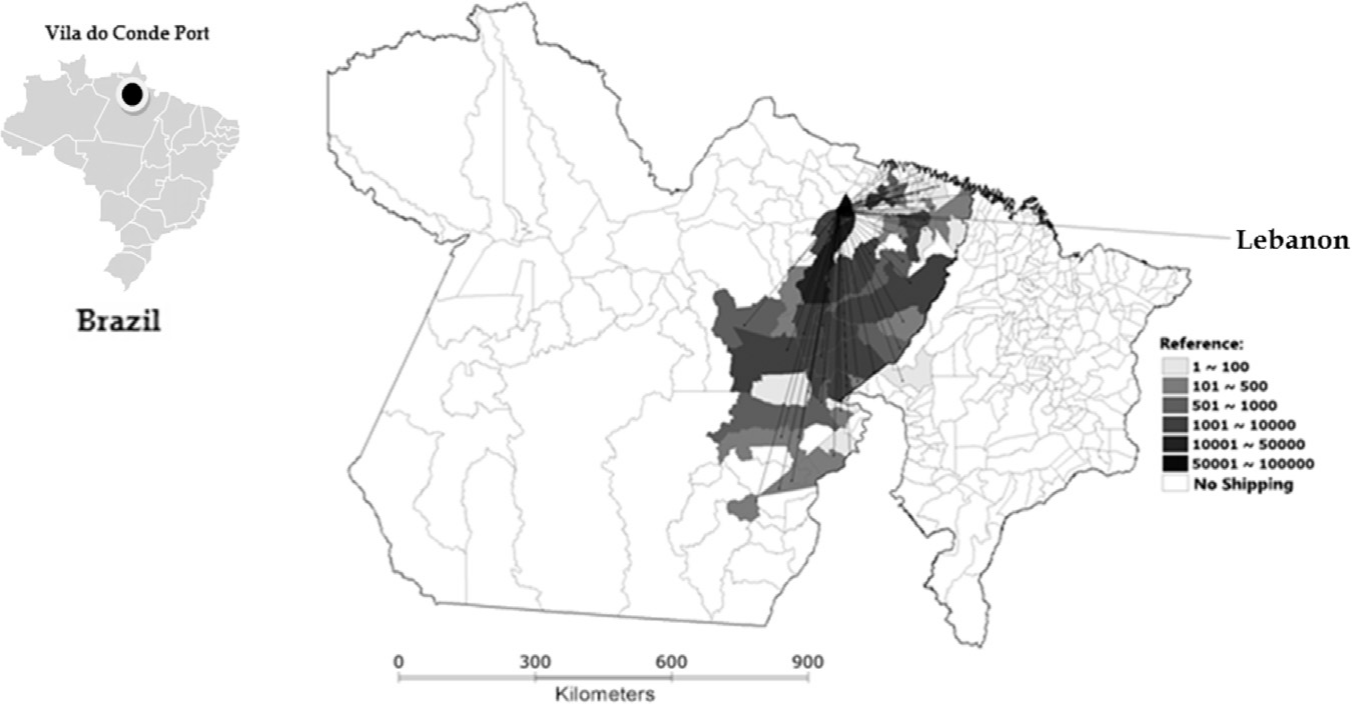
Flow of live cattle exports from Vila do Conde Port to Lebanon by municipality and number of animals, from Pará and Maranhão states in 2009 (with adaptations).

**Fig. 4:**
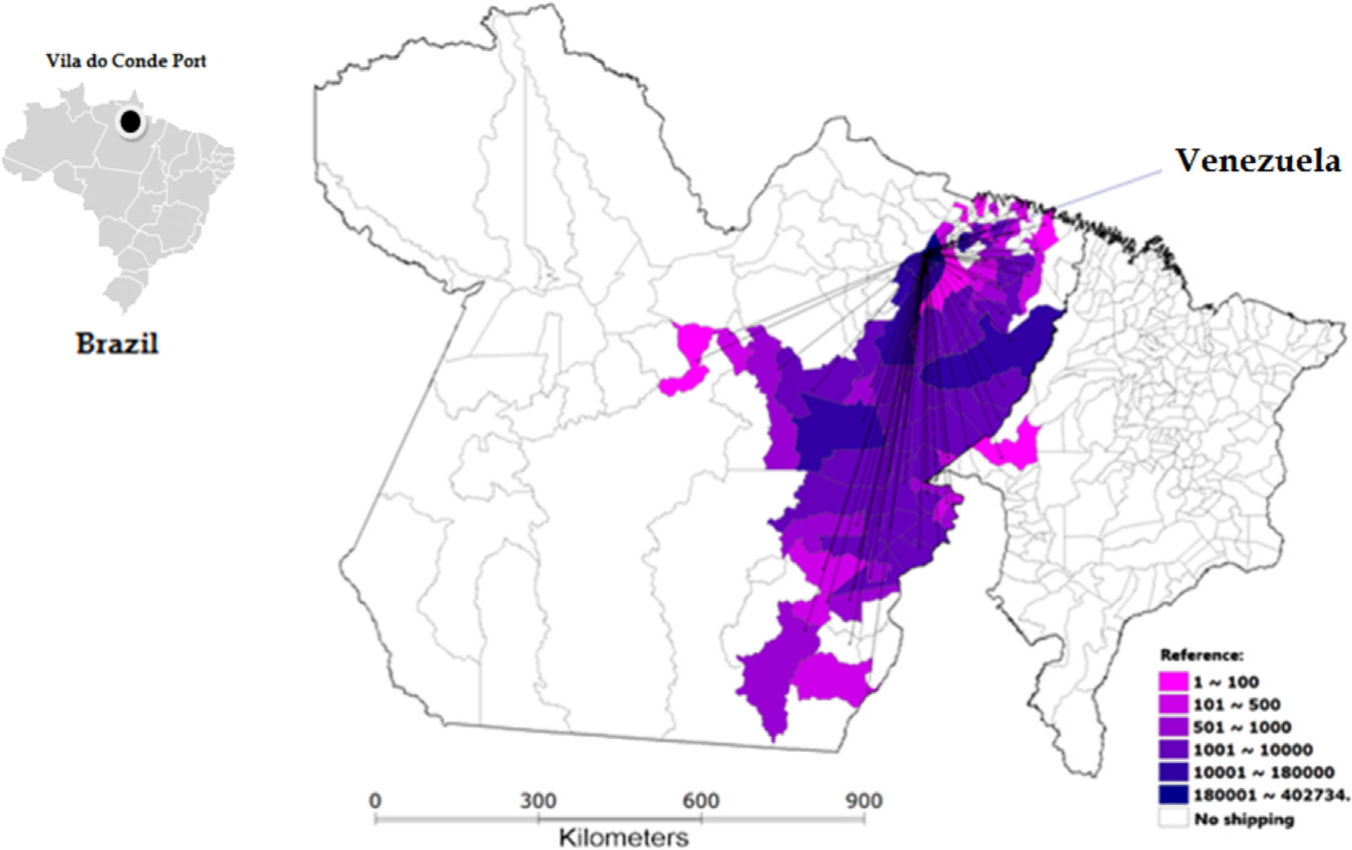
Flow of live cattle exports from Vila do Conde Port to Venezuela by municipality and number of animals, from Pará and Maranhão states in 2009 (with adaptations).

Ten different municipalities in Pará exported more than 10,000 live cattle each. In addition, four towns in this state (Mojú, Igaparé - Miri, Abaetetuba and Paragominas) provided in total 294,552 animals, representing 59.2% of all cattle that embarked on Vila do Conde Port. Networks of live cattle export from Vila do Conde Port depending on the destination country can be seen in Fig. 2, Fig. 3, Fig. 4. A total of 6242 animals from 12 municipalities, especially Marabá (Pará state) and Araguaína (Tocantins state), were loaded for Egypt, with 64.8% of all cattle (Fig. 2) being exported to this country. Sixty municipalities exported 94,850 animals to Lebanon and four (Moju, Castlebay, Paragominas and Igarapé-Miri, all in Pará state) concentrated 55.1% of cattle export to that country (Fig. 3). For Venezuela, 402,734 animals from 71 municipalities were exported, with Abaetetuba, Moju and Igarapé-Miri in Pará accounting for 53.6% of cattle exports (Fig. 4).

**Fig. 5:**
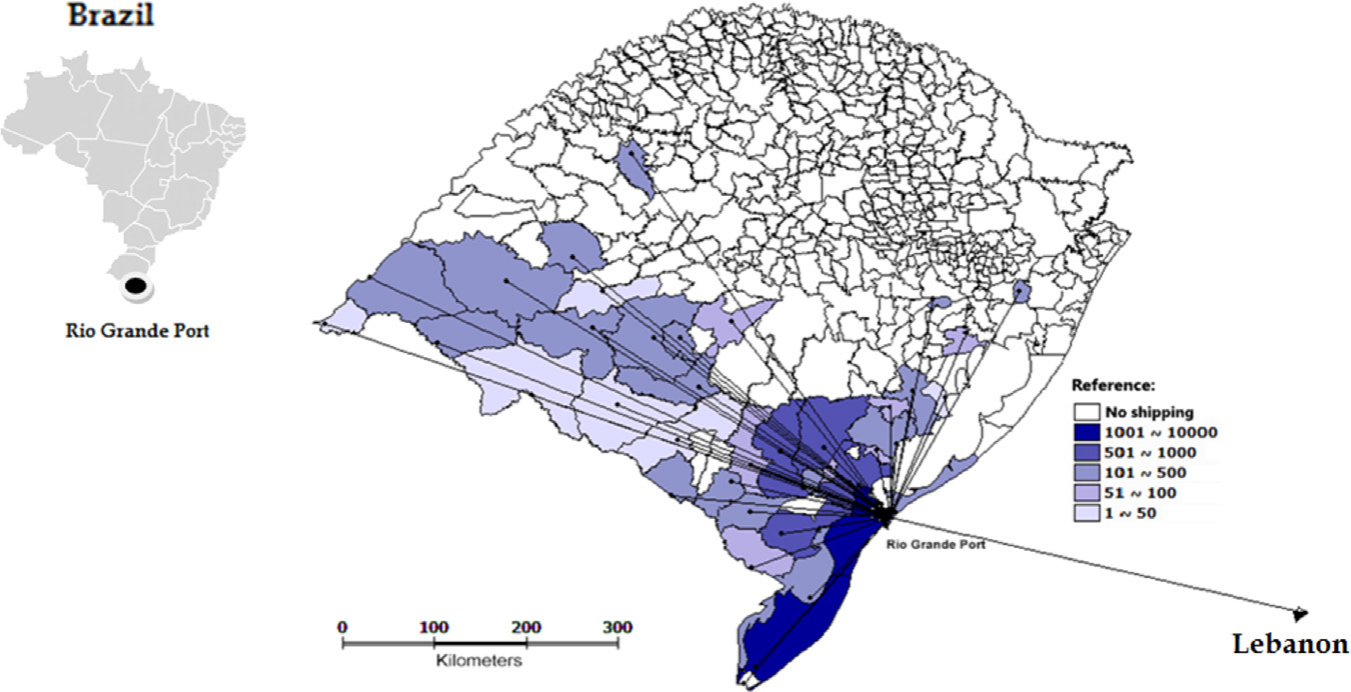
Flow of live cattle exports from Rio Grande Port to Lebanon, by municipality and number of animals, from Rio Grande do Sul state, in 2009 (Terraview® 4.2.0 - with adaptations).

**Fig. 6:**
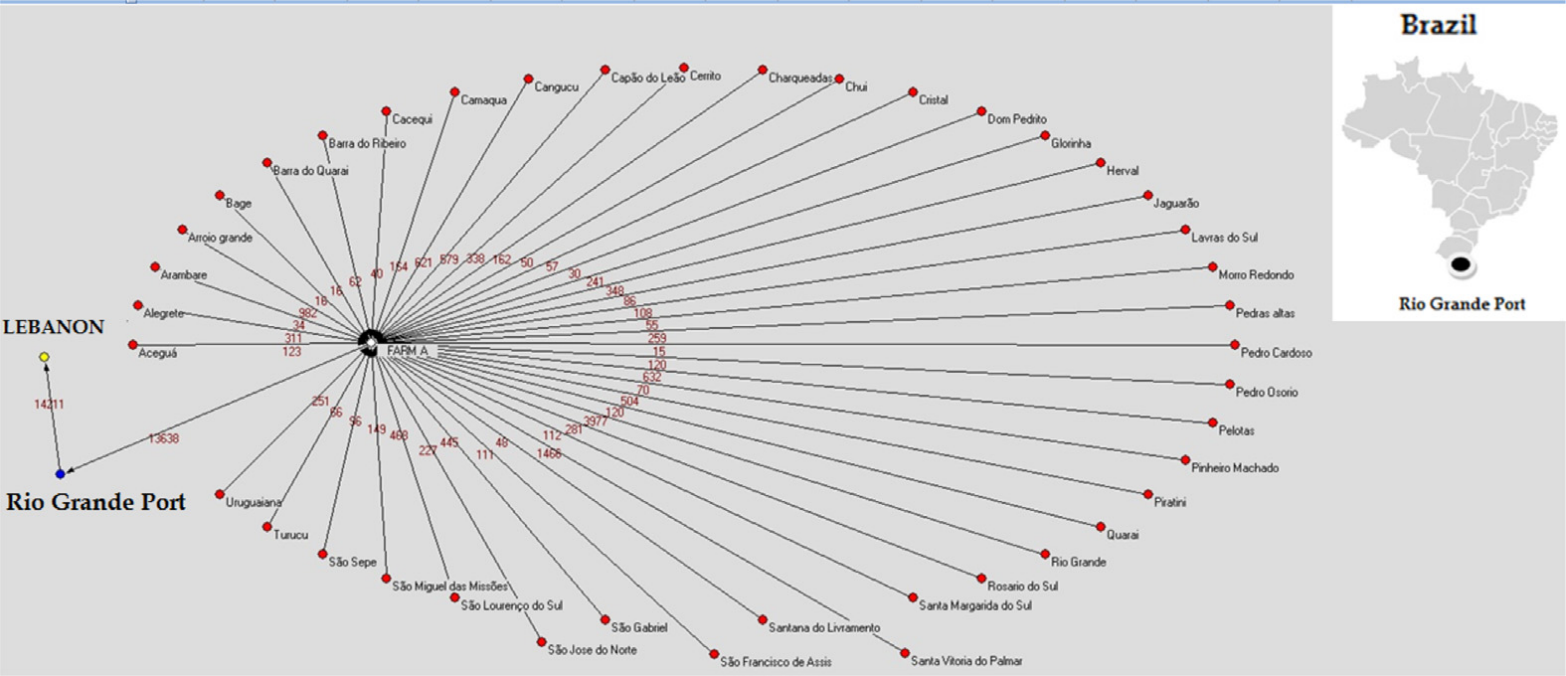
Flow of live cattle exports from Rio Grande Port to Lebanon, by municipality and number of animals, from Rio Grande do Sul state, in 2009 (Pajek® 6.4 - with adaptations).

Cattle exported through the Rio Grande Port came from 236 farms located in 42 municipalities of Rio Grande do Sul state. Among these, the most important municipalities were Rio Grande, with 42 farms and 3932 animals, Santa Vitória do Palmar (26 farms and 1466 animals) and Arroyo Grande (18 farms and 982 animals). In the municipality of Rio Grande, a single establishment, identified as “Farm A” was responsible for the concentration of animals, where animals were assembled and sent to Lebanon. This property, only five miles away from the Rio Grande Port, received 13,838 animals originated from 229 farms located in 41counties within the state. The flow of cattle exported through the Rio Grande Port is shown in Figs. 5 and 6. A total of 14,211 cattle was sent to Lebanon, of which 13,638 (96%) were from Farm A and 573 (4%) from seven other farms. Farm A and the Rio Grande Port were not distinguished on the map, due to the proximity between them, which prevented individual geographical representation on the scale used.

All live cattle exported from São Sebastião Port to Angola, came from a single property located in the municipality of Castilho, São Paulo state. Cattle exports from VCP bound for Venezuela also came from a single property located in Uberaba city, Minas Gerais state. Google Maps® (Google Inc, Mountain View, CA) generated corridors and suggested 83 (93.2%) of the 89 shortest distances between the cities of origin contained in the ATCs and Barcarena city. Therefore, 477,245 (92.9%) of live cattle exported through Vila do Conde Port probably used these corridors. The shortest distance between the cities of origin and Vila do Conde Port was 53 km, referring to the path from Abaetetuba to Barcarena cities. The largest distance was identified from Curuçá to Barcarena municipalities (1823 km). The mean distance for the shortest corridor between farm and port suggested was 477.45 km. If the corridors listed were in fact those used, an estimated average number of animals transported per path was of 5596, with an average of 18.76 animals per ATC issued. The cattle from Tocantins, Maranhão and Pará states travelled on average 821,565 and 420 km, respectively, to reach Barcarena city.
